# An injectable CRISPR therapy instructs B cells to produce anti-HIV antibodies

**DOI:** 10.1093/synbio/ysac027

**Published:** 2022-11-08

**Authors:** Logan Thrasher Collins

**Affiliations:** Department of Biomedical Engineering, Washington University in St. Louis, St Louis, MO, USA

Although the immune system is well known as the guardian of the human body, certain infections and cancers can overwhelm its protective barriers. Over the past decade, scientists have developed genetic engineering tools that can enhance our immune systems to the point where they overcome such difficult threats. One success story in this area is the use of chimeric antigen receptor T cell (CAR T) therapy for blood cancers (1, 2). CAR T cells are engineered immune cells programmed to detect and destroy the cancer. In order to reprogram T cells, CAR T therapies require taking a blood sample out of a patient, shipping the sample to a laboratory, genetically modifying T cells within the sample, purifying the modified T cells, shipping them to the hospital and injecting them back into the patient. The cost, slowness and complexity of engineering immune cells outside of the body have limited accessibility of CAR T therapies and have challenged the expansion of this technology to the engineering of other immune cells such as B cells (3–5). To help overcome these barriers, a recent study was performed in Adi Barzel’s laboratory at Tel Aviv University and published in *Nature Biotechnology*. Nahmad *et al*. developed an injectable Clustered Regularly Interspaced Short Palindromic Repeats (CRISPR)-based gene therapy to directly modify B cells inside of the body, giving them the ability to produce an antibody that fights acquired immune deficiency syndrome (AIDS) infections (6). In the future, such an injection might make immune cell therapies cheaper and thus more accessible to everyone and may pave the way for a vaccine against AIDS or a potent treatment for people who already suffer from the disease.

CRISPR acts as a biomolecular cut-and-paste system that can insert genetic instructions at desired locations within the genome. It uses a protein–RNA complex consisting of a Cas9 protein and a guide RNA (gRNA) to cut a sequence within the genome that is recognized by the gRNA. After the cut has been made, one can provide a new piece of DNA instructions that the cell will stitch into the cut site during repair. CRISPR makes genetic alteration of cells much easier by precisely targeting where to put new DNA into the genome.

Nahmad *et al*. injected mice with engineered adeno–associated viruses (AAVs) for delivery of (i) a gene encoding an anti–human immunodeficiency virus (HIV) antibody and (ii) genes encoding CRISPR Cas9 and gRNA machinery. AAVs act as a type of delivery system for transporting DNA into human cells and are commonly used in gene therapy. To ensure that the CRISPR genes would only activate when the AAVs were taken up into B cells, a promoter (a genetic on–off switch) that selectively turns on within B cells was employed to drive the expression of the CRISPR system. This minimized toxicity by greatly decreasing CRISPR-mediated cutting of genomic DNA in non-target cells such as liver hepatocytes.

Nahmad *et al*. designed their Cas9-gRNA complex to facilitate the insertion of the anti-HIV antibody gene at a site within the B cell genome called the immunoglobulin heavy chain locus. B cells use this genomic locus to generate diverse variations of antibodies in response to continuously evolving infections, so this locus allows the body to modify its antibodies to keep up with the evolution of pathogens. Although the authors did not explore such an evolutionary arms race experimentally, they did show that the mice’s B cells generated variations of the original anti-HIV antibody gene, indicating that such a feature may work successfully in the future.

Injectable viruses that engineer B cells inside the body via CRISPR circumvent the need for complicated *ex vivo* steps, lowering the cost of emerging immunotherapies. Nahmad *et al.*’s proof of concept is an exciting advance for making B cell immunotherapies more translatable and accessible and illustrates how synthetic biology is pushing the boundaries of medicine toward a world with vaccines and cures for currently terrible illnesses like cancer and AIDS.
